# The Functions of Metamorphic Metallothioneins in Zinc and Copper Metabolism

**DOI:** 10.3390/ijms18061237

**Published:** 2017-06-09

**Authors:** Artur Krężel, Wolfgang Maret

**Affiliations:** 1 Department of Chemical Biology, Faculty of Biotechnology, University of Wrocław, Joliot-Curie 14a, 50-383 Wrocław, Poland; artur.krezel@uwr.edu.pl; 2 Division of Diabetes and Nutritional Sciences, Department of Biochemistry, Faculty of Life Sciences and Medicine, King’s College London, 150 Stamford Street, London SE1 9NH, UK

**Keywords:** metallothionein, thionein, zinc, copper, metamorphic proteins, affinity

## Abstract

Recent discoveries in zinc biology provide a new platform for discussing the primary physiological functions of mammalian metallothioneins (MTs) and their exquisite zinc-dependent regulation. It is now understood that the control of cellular zinc homeostasis includes buffering of Zn^2+^ ions at picomolar concentrations, extensive subcellular re-distribution of Zn^2+^, the loading of exocytotic vesicles with zinc species, and the control of Zn^2+^ ion signalling. In parallel, characteristic features of human MTs became known: their graded affinities for Zn^2+^ and the redox activity of their thiolate coordination environments. Unlike the single species that structural models of mammalian MTs describe with a set of seven divalent or eight to twelve monovalent metal ions, MTs are metamorphic. In vivo, they exist as many species differing in redox state and load with different metal ions. The functions of mammalian MTs should no longer be considered elusive or enigmatic because it is now evident that the reactivity and coordination dynamics of MTs with Zn^2+^ and Cu^+^ match the biological requirements for controlling—binding and delivering—these cellular metal ions, thus completing a 60-year search for their functions. MT represents a unique biological principle for buffering the most competitive essential metal ions Zn^2+^ and Cu^+^. How this knowledge translates to the function of other families of MTs awaits further insights into the specifics of how their properties relate to zinc and copper metabolism in other organisms.

## 1. A 60-Year Old Conundrum about a Protein’s Function

The story of metallothionein began with the discovery of a cadmium-containing protein in horse kidneys in the 1950s [1]. The name metallothionein (MT) originated from the protein’s variable metal content and its relatively high amount of sulphur [2]. Cadmium accumulates in MT with exposure and age. However, the metal ions most relevant biologically are zinc (Zn^2+^) and copper (Cu^+^). A charge difference—later shown to be due to the presence or absence of the amino acid Asp at position 10—allowed separation of two major forms by ion exchange chromatography and led to the designation of them as MT1 and MT2. *MT1* in most mammalian species has many related genes (paralogues) and the resulting proteins can be resolved on reversed phase HPLC columns [3]. In an unrooted evolutionary tree, MT2 groups closely with MT1 proteins, which have at least 8 functional genes in humans [4]. Two additional forms, MT3 [5], a neuronal growth inhibitory factor, and MT4 [6], present in squamous epithelia, were found much later and are evolutionarily more distantly related. Horse kidney MT1B was sequenced twenty years after the discovery of the protein [7]. The sequence showed characteristic spacings of cysteines and the absence of aromatic amino acids. It took yet another 10+ years to obtain 3D structures of hepatic MTs with both X-ray crystallography (rat MT2), showing the entire molecule, and NMR spectroscopy (human MT2), showing the individual domains but not how they interact [8,9]. The peptide chain folds into two separate domains, each organizing two Zn^2+^–sulphur(thiolate) clusters, one with 3 Zn^2+^ and 9 Cys (β-cluster) and the other with 4 Zn^2+^ and 11 Cys (α-cluster). In both clusters, all metal ions are in tetrathiolate coordination environments, which is possible only through the use of cysteine sulphur as a donor bridging two metal ions. It is important to realize that these structures are based on either a species induced in vivo with Cd^2+^ [9], which remarkably has two Cd^2+^ ions in the N-terminal β-domain at defined positions, or a species where all the metal ions had been removed, all the cysteines reduced, and the protein was reconstituted with seven Zn^2+^ or Cd^2+^ ions. In vitro, MTs can bind more than seven equivalents of metal ions [10,11]. Thus, for structural studies the proteins were brought into chemically defined “homogeneous” forms—a requirement for most biophysical methods to obtain molecular structure—whereas the inherent heterogeneity of the protein in vivo provides a clue to its functions. It is therefore critical to understand that the forms for which structures exist do not present the dynamic state of the protein in vivo.

Metalloproteins with multiple and similarly spaced cysteine residues were found in many non-mammalian species and are also called metallothioneins or metallothionein-like [12]. Sequence similarity and information on genomic DNA such as introns, exons, and the presence of *cis* acting elements formed the basis for a classification of metallothioneins into 15 families [13]. 8156 entries (1657 nucleotide sequences and 6499 expressed sequence tags (ESTs) for MTs from various species were in the National Center for Biotechnology Information (NCBI) nucleotide database in April 2017. In addition, classification of plant MTs, which have significant variability in primary structures, required the definition of four families [14]. It is important to acknowledge that the common evolutionary origin of MTs has not been established, however. Taxonomic distribution has been reported but not phylogenetic relationship based on a rooted tree. This lack of information is problematic if the term metallothionein is used to imply a functional relationship across phyla—a fallacy in many scientific reports. Furthermore, the 3Zn^2+^–9Cys (Zn_3_S_9_) cluster of the β-domain of MT is found in the SET domain of histone lysine methyltransferases [15] and the CXC domain of male-specific lethal 2 protein [16], clearly demonstrating a functional context of the metal-binding motifs outside the MT family. Many criteria have been discussed to define what constitutes a metallothionein, but for any of them exceptions were found. The definition, therefore, falls back to what the name initially was meant to express: proteins that have metal ions and thiol(ate) sulphur. It must be understood that the name does not describe a function but a generic property like, for example, the cysteine knot proteins. To avoid generalizations about MTs in other phyla, we will focus here only on human MTs, with occasional reference to other mammalian MTs as appropriate, necessary, and indicated. We believe that a deductive approach to determine biological function is flawed in the case of the accumulated sequences of so-called MTs and that an inductive approach is necessary instead.

“Metallothionein” is a vast and multifarious subject. With only properties of the protein known, how can one find out its natural function(s)? The search for a function has been on-going for over 60 years and reached a nadir when the phenotypes of *Mt1/Mt2* k.o. mice indicated that these proteins are not essential under laboratory conditions and accordingly the function was thought to be elusive [17,18,19]. However, in these experiments the phenotype with regard to zinc metabolism was never tested, and when it was tested later, a lot of important observations were made, all of which in fact do point to specific functions [20]. Many genetic polymorphisms of human MTs have been described. Some of them result in mutations in the proteins that are associated with multiple effects on human health and also point to specific biological roles of MTs [21].

The generally quoted functions of MTs are believed to be in the metabolism of toxic metal ions, i.e., Cd^2+^, the metabolism of essential metal ions, i.e., Zn^2+^ and Cu^+^, and as antioxidants or radical scavengers. One function focuses on metal ion binding and the other focuses on thiol reactivity, while in fact the two are linked with important consequences. Two developments, we believe, require re-evaluating the basic tenets of structural studies as they provide clues to the functions of MTs. One development is information about metal ions binding to MTs in vivo and the other is about the affinity of metal ions for the protein, the most important property for its function in metal metabolism. At issue is the assumption that the form with 20 reduced cysteines and seven Zn^2+^ ions made in vitro and used for deriving structural models is the only form of the protein. As indeed noticed early on, in vivo MTs have variable metal ion content under different conditions (Table 1), and even for a single metal ion such as Zn^2+^ the occupancy is variable. Also, an antioxidant function implies different redox states of the protein, for which there is evidence as well. For example, under conditions of oxidative stress MT containing a disulphide bridge has been isolated [22]. By using rapid chemical modification assays, we have shown that the protein in cultured human cell lines is neither saturated with Zn^2+^ nor fully reduced [23,24].

The two properties are linked: Metal occupancy determines thiol reactivity and thiol reactivity determines metal ion binding characteristics. The resolution of the underlying conundrum of apparently different functions is that the Zn^2+^-thiolate clusters are redox-active in the cellular environment. It allows coupling the redox-inert Zn^2+^ ion to redox metabolism by using redox-sensitive MT to provide more Zn^2+^ under oxidative conditions and less Zn^2+^ under reductive conditions, thus establishing a role for redox changes in zinc availability and distribution [28,29]. The term “metallothionein” (MT) refers to several possible forms of the same protein, which differ with regard to the bound elements, metal load and redox state. To avoid confusion due to this generic nature of the term, we will use “metallothionein” or “MT” to refer to a particular protein isoform with a unique amino acid sequence without considering its native heterogeneity. Whenever possible, we will additionally specify the particular states in terms of metal load and oxidation state, using a nomenclature that identifies the molecular species relevant to function.

While the affinity of MT for Zn^2+^ is high overall, originally reported binding constants were estimates only and the methods used at the time did not have the resolution to determine small differences in affinities of particular metal ions in such a complex system. When we investigated the affinities with higher resolution methods, we found a distribution of affinities and binding of one Zn^2+^ ion with lower affinity in human MT2A [30]. This is chemically perhaps not remarkable: Though the Zn^2+^ ions are all in tetrathiolate coordination environments, there are differences in the number of bridging and end-on ligands, and in neighbouring amino acid residues of the metal-binding cysteine sulphurs. The best demonstration that the seven coordination environments are different is provided by ^113^Cd or ^111^Cd-NMR spectroscopy which can resolve all seven metal ions into individual peaks [31,32]. But it is remarkably for biology that the difference in affinities rules out that MT is a thermodynamic sink for cellular Zn^2+^, i.e., a protein in which Zn^2+^ ends up due to binding with the highest affinity among all zinc proteins. Our findings support the opposite function, namely the active participation of MT in Zn^2+^ re-distribution. Quantitative investigations of zinc affinities of other proteins now confirm that MT does not have the strongest Zn^2+^ binding sites [33]. Other proteins, frequently with mixed S_2_N_2_, S_3_N coordination environments, such as the PDLIM1 protein and transcription factors with a classical ββα fold (log*K*_b_ ≈ 14) and the intermolecular zinc hook site of the Rad50 protein (femtomolar affinity) bind Zn^2+^ ions much more tightly than MT, putting MT in the middle of the action rather than at its periphery [33,34,35,36].

Significantly, all postulates and speculations about the functions of the protein in metal metabolism made in the last sixty years have been in vain because the molecular aspects of the control of cellular zinc homeostasis were not known. Only now with new knowledge gradually emerging in the last 10+ years on how cellular Zn^2+^ is regulated, a role of MTs in zinc metabolism can be formulated. It is now established that Zn^2+^ is bound with rather high affinity to cellular zinc proteins and that the availability of free Zn^2+^ ions accordingly is very low. In chemical terms, this means that Zn^2+^ is buffered at high pZn (−log([Zn^2+^]_free_) and Cu^+^ even at higher pCu (−log([Cu^+^]_free_). There is no storage for Zn^2+^ in a protein akin to ferritin for Fe^3+^. Instead there are controlled (gated) vesicular stores for Zn^2+^ in the cell. For efficient buffering, there needs to be a surplus of coordination sites that are not occupied with metal ions [23]. In this regard, the properties of MT are significant. Its affinity for Zn^2+^ matches cellular pZn values and it has unoccupied metal-binding sites available for buffering. The term “unoccupied” has a special meaning in the case of MT. It does not necessarily refer to donors of ligands not bound to a metal ion such as in a metal-depleted active site of a metalloenzyme. Instead, it refers, at least in part, to the additional metal binding capacity during the transition of isolated tetrathiolate coordination environments with only end-on ligands—a maximum of five Zn^2+^ ions bound to twenty cysteines—to the clusters with bridging ligands—seven Zn^2+^ ions bound to twenty cysteines. In addition, for copper buffering, it refers to one metal ion (Cu^+^) displacing a less competitive metal ion (Zn^2+^) in an already occupied site. These inherent properties of MTs are indeed remarkable features of a uniquely biological metal-buffering molecule. An even and specific stoichiometric metal/ligand ratio is a chemical concept with limited applicability to biology, because in vivo the availability of metal ions is controlled and dictates stoichiometric or even non-stoichiometric ratios. Also, it is now established that Zn^2+^ ions have signalling functions akin to Ca^2+^ ions [37]. This is the second reason why a molecule such as MT with fast Zn^2+^ binding and dynamic regulation is needed for buffering signalling zinc ion transients. Thus, while we are beginning to realize the intricacies of cellular zinc regulation, an understanding develops on how the properties of MTs relate to functions in zinc metabolism. In retrospect, one realizes that a function of MT in zinc or copper metabolism could not have been foreseen earlier because it was not known how these metal ions are regulated and the molecules regulating them were not even known. Only now the affinities of MTs for Zn^2+^ can be understood in terms of how Zn^2+^ is regulated, i.e., buffering both steady state and transient levels of Zn^2+^. MT clearly fulfils a very important function. Research on the detailed MT functions in zinc and copper metabolism is just beginning and will intensify in the future with a concomitant understanding of zinc fluxes and transients in real time and cellular space, and how differences in affinity, redox potential, and differences among MT isoforms control Zn^2+^ in different tissues.

Many scientific articles state that the function of MT is elusive or enigmatic. Yet the potential functions and the functional potential of mammalian MT in zinc metabolism have been discussed repeatedly [38,39]. The full implications of a role in zinc metabolism could not have been known at that time because the two dozens of zinc transporters that participate in the control of cellular zinc homeostasis and the concept of signalling Zn^2+^ ions were not known. While the protein’s functions point to Zn^2+^ (and Cu^+^) metabolism, the regulation of the *MT* genes also point to Zn^2+^ metabolism, and this latter fact also has not been discussed in sufficient detail. The regulation of gene expression of MTs by many factors is a most dynamic system, exactly what is needed for dealing with such a critical cellular ion as Zn^2+^, and it adds significant additional support for a role of the mammalian MTs in zinc metabolism. Clearly, making this connection for non-mammalian MTs will require additional information on their properties in relation to the specifics of zinc and copper metabolism in the particular organisms.

## 2. Relationships among Human Metallothioneins

An unrooted phylogenetic tree of human MTs shows a clear separation of MT3 and MT4 from the other MTs that branch out [40]. In the branch, MT2 is close to MT1G and F, i.e., MT2 and MT1 are much more closely related to each other than to MT3 or MT4. MT2 is a member of the MT1 branch. The multiplicity of MT1 forms in humans has been observed in many other mammalian species but apparently not in mice [41]. The reason for this gene multiplication is not clear. It is also not entirely clear how many functional human MT1 proteins exist. Fourteen tightly linked *MT* genes (*MT2A* and thirteen *MT1* genes, including six genes not previously described) were localized on human chromosome 16q13 [42]. In the order the *MT1* genes occur on the chromosome, they are named 1L, 1E, 1K, 1J, 1A, 1D, 1C, 1B, 1F, 1G, 1H, 1I and 1X. Upon further examination of the six novel genes (1I, 1J, 1K, 1L, 1H, 1X), it was concluded that only 1H and 1X are functional genes while the others, including 1D, are pseudogenes [42,43]. Some authors [40] agree with us [4] on MT1A, MT1B, MT1E, MT1F, MT1G, MT1H, and MT1X being functional proteins, but list MT1K (also known as MT1M) as expressed and *MT1L* (also known as MT1R) as a pseudogene [4]. Minimally, there are eight MT1s plus MT2, MT3 and MT4, i.e., 11 functional proteins. The branch that contains closely related MT2, MT1G, and MT1F is more distantly related to MT1H, MT1X. Another branch contains MT1A, MT1E, MT1B, MT1K (MT1M), and *MT1J* (pseudogene). There is an urgent need to curate databases and agree on the nomenclature. Without agreement on a function of MTs, of course, it is difficult to make a judgement as to whether or not a particular form is functional. An assignment as a pseudogene, in part, rests upon the occurrence of amino acids that are atypical for MTs. Table 2 presents UniProtKB/Swiss-Prot entries, as well as annotation scores of MT isoforms along with their natural variants. This list differs from the one given above [40] where *MT1L* (MT1R) is listed as a pseudogene.

## 3. Galvanization of Human Metallothioneins’ Gene Expression

**Figure 1:**
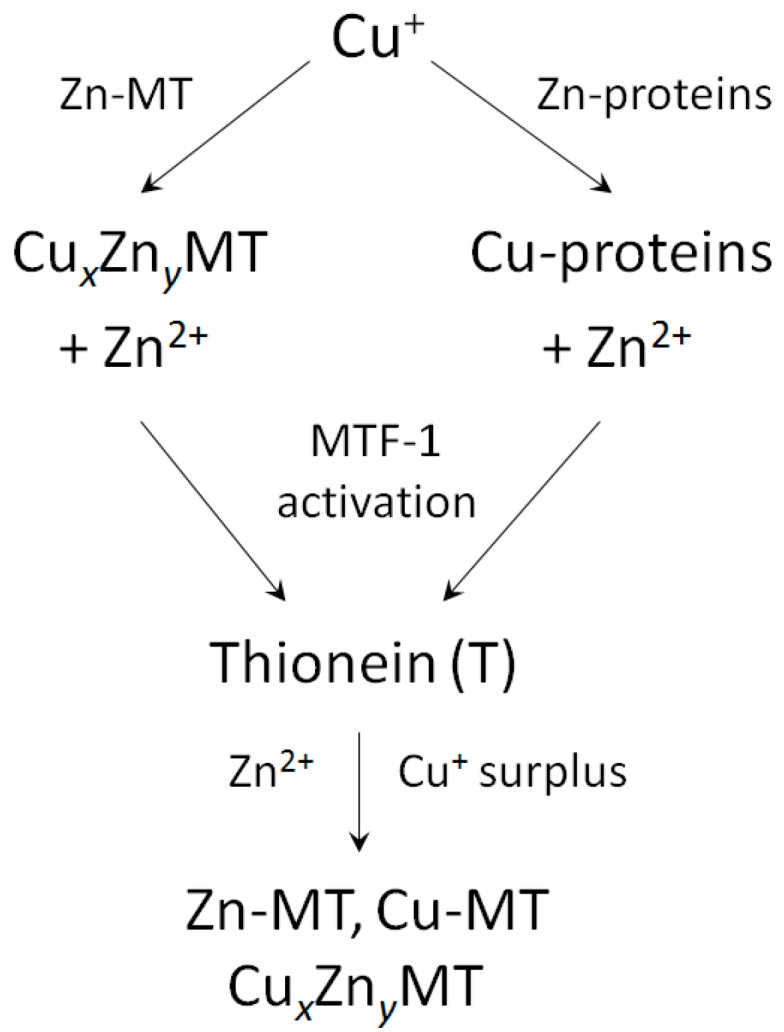
Crosstalk between Cu^+^ fluxes and zinc proteins (including Zn-MT). Released Zn^2+^ activates the metal-response element (MRE) binding transcription factor-1 (MTF-1) transcription factor, which promotes biosynthesis of thionein (apo-metallothionein). In vivo-synthesized thionein interacts with released Zn^2+^ and Cu^+^ surplus forming metamorphic forms of metallothioneins.

In contrast to single functional genes for *MT2*, *3* and *4*, multiple functional genes for *MT1* are present in the human genome [42,43]. A plethora of factors and conditions control basal and induced expression of MTs. In addition, gene expression is controlled by promoter methylation and histone modifications, where zinc-dependent proteins and events play a role. The human *MT2* (*MT2A*) promoter has *cis*-elements for binding of the transcription factor Sp1 (a zinc finger protein), eight sites for binding of MTF-1 (metal-response element (MRE) binding transcription factor-1, a zinc finger protein), one site for AP1 binding, overlapping with one for AP4, three sites for AP2 (binding sites for activator proteins—transcription factors responsive to different signalling pathways), a glucocorticoid responsive element binding proteins from the nuclear hormone receptor family, which have zinc twist motifs, and an interferon-sensitive response element for interferon alpha signalling [44]. The human *MT3* promoter includes sites for binding of Sp1, AP-2, MTF-1, and an astrocyte-specific transcription factor [45]. Human MT4 expression is developmentally regulated. The following summary is based on experimental evidence and in silico analyses of the promoters of human *MT1* genes [40]. Zinc features prominently in basal transcriptions factors (Sp1, MTF-1), in many of the transcription factors involved in the induction (Table 3), and in repressors of *MT* gene expression. Zinc binding includes different types of zinc motifs: zinc fingers (1Zn^2+^), zinc twists (2Zn^2+^) and zinc clusters (2–3Zn^2+^). Multiple *cis*-elements are present in the genes of the MT1 family (Table 3). With few exceptions, at least seven Zn^2+^-dependent proteins are involved in the induction of a particular MT. MTF-1 is a basal transcript factor and also involved in the induction of MT depending on which and how many MRE *cis*-elements on the promoter it uses, and its repression. If one adds up the Zn^2+^ ions needed for e.g., induction of MT1A, assuming that all *cis*-elements are used, one calculates that 30 Zn^2+^ ions are present in all the transcription factors. In such an analysis of gene expression, only the downstream Zn^2+^-dependent transcription factors are examined. Of course, additional Zn^2+^-dependent events occur upstream in the signal transduction pathways to these transcription factors. MT itself is involved in activating MTF-1. For example, cadmium-induced MT expression proceeds through Cd^2+^ displacing Zn^2+^ in MT and the released Zn^2+^ then activating MTF-1 [46,47]. MT also modulates glucocorticoid responsiveness of cells [48]. Thus MT participates in controlling the occupancy of Zn^2+^-dependent transcription factor with Zn^2+^ either through its role in buffering Zn^2+^ or in delivering Zn^2+^ to the transcription factors directly. A specific sensor for copper has not been identified in eukaryotes. Like Cd^2+^, Cu^+^ displaces Zn^2+^ from MT and the released Zn^2+^ then induces thionein through MTF-1 in order to buffer any surplus of Cu^+^ (Figure 1).

Four negative regulators have been identified for *MT2A*: MTF-1, NF-I/CTF, which binds to a CCAAT *cis*-element, PZ120 = ZBTB11, and C/EBPa (bZip motif) [38]. The PZ120 protein has two types of zinc motifs, the poxvirus and zinc finger (POZ) motif and additional zinc fingers. The protein lacking the zinc finger domain serves as a repressor, indicating other functions of these domains [44].

There are significant differences in the promoters of the genes of the *MT1/MT2* branch (Table 3).

In part, these differences relate to tissue specific expression of MTs. The underlying differences have not yet been linked with any tissue specificity of zinc metabolism. Nor has the vast information about differential gene expression of MTs in cancer and other pathological conditions been explained in terms of specific effects on zinc or copper metabolism. Analysis of mRNA levels or protein levels—if suitable antibodies are available—provides no information on either the redox state or the metal load of the protein. Because *MT* gene expression is very sensitive to pathological changes in addition to many forms of stress, and environmental and developmental changes it could serve as a surrogate for yet to be defined changes in metal and redox metabolism under these conditions. Suffice it to say that observed changes in gene expression of MTs in cancer cells are entirely consistent with de-differentiation and proliferation of cells and the critical roles Zn^2+^ has in these processes.

## 4. Function in Human Cellular Zinc Metabolism

Gene expression studies cannot answer the question how the function of the expressed MTs relate to redox and metal metabolism and to donating or accepting Zn^2+^/Cu^+^. As for many proteins there is very poor correlation between *MT* mRNA level and MT protein level [49]. Here, too, using a consistent nomenclature would improve our understanding. While most authors use the term metallothionein for the expressed protein, the protein made on the ribosome is thionein (T), not metallothionein. Whether newly synthesized T becomes MT depends on the availability of metal ions, which in the case of the highly competitive Zn^2+^ and Cu^+^ ions is very limited and highly controlled. MT is a metal donor while T is a metal acceptor. Functionality of the two protein forms therefore is opposite and can be determined only with regard to information about metal occupancy on the protein level. But establishing functionality of the individual MTs is almost never pursued with assays that determine metal load and redox state. We have developed such assays for determining the overall metal load of all MTs present in a cell or tissue, i.e., the MT/T ratio [50]. The value of this ratio determines zinc availability. At high ratios, more Zn^2+^ is available; at low ratios, less Zn^2+^ is available as expected for a metal buffer. In rat organs, the percentage of apoprotein (T/(T + MT)) varies from 9% in testes to 53–54% in brain and kidneys [50]. In vitro investigations demonstrated that varying the MT/T ratio indeed controls the potential of MT to serve as a donor of Zn^2+^ for apoproteins [51]. Changes in MT expression affecting the overall amount of the protein are also important. They adjust the buffering capacity of the cell. Buffering capacity is the second property of a buffer and different from adjusting a particular pZn. It determines how resistant the system is to a change in pZn when the total Zn^2+^ concentration changes. Altering metal buffering is critical for the physiology of a cell. Different states of the same cell, i.e., proliferating, differentiating, and apoptotic cells have different zinc buffering and accordingly different pZn values [23].

High Zn^2+^ ion concentrations induce T to bind and buffer Zn^2+^ (and Cu^+^). How the cell senses when its Zn^2+^ concentration is too low is a different matter and not resolved. The induction of T at high Zn^2+^ ion concentrations seems to relate mostly to conditions of binding an excess of metal ions, which is a function of T in accepting metal. An important issue was to demonstrate that T exists under steady state conditions and in which form, i.e., whether it is entirely in the apoform or a protein that contains less than seven metal ions. Whether T can co-exist with fully-loaded MT depends on whether there is a high degree of positive cooperativity in metal binding promoting the co-existence of the two species. The issue of cooperativity will be addressed later. In many cancer cell lines, evidence for T was provided [52]. We addressed the question of whether T exists in tissues by employing a rapid chemical modification of thiols. The results provided further evidence that the protein is not fully saturated with metal ions, exactly what one expects from a metal buffer [50]. However, an important distinction must be made when using the term thionein (T). The completely metal-free form of the protein can be made in vitro but based on the metal binding properties and the availability of metal ions in biological systems T does not exist in vivo*,* with the possible exception of its fleeting existence when it is made on the ribosome.

It is now established that the cellular Zn^2+^ concentration is tightly regulated by zinc transporters, exporters and importers and that transient increases in cellular Zn^2+^ concentrations are employed for cellular signalling. The individual molecules controlling cellular zinc homeostasis, including MT, are interconnected and do not work in isolation. This information was not available twenty years ago when the function of MT was deemed elusive. Regarding mobilization of Zn^2+^ for signalling purposes there are at least three different pathways [53]: (i) release of Zn^2+^ from cells by vesicular exocytosis, which has to be followed by “soaking up” the Zn^2+^, e.g., in the synaptic cleft; (ii) the release of Zn^2+^ at the endoplasmic reticulum by hormone-triggered phosphorylation of the zinc transporter ZIP7 [54]; and (iii) Zn^2+^ release through signalling with reactive species such as hydrogen peroxide, nitric monoxide or yet others from redox-sensitive thiolate coordination environments. These events generate different spatiotemporal distributions of signalling zinc ions. As with calcium ions, the steady state needs to be re-established after the signalling event occurred. MT indeed influences these zinc signalling events in the cell [55] and therefore its expression is a major factor in modulating and controlling zinc-dependent cellular signalling.

## 5. Metal Composition: Native Mammalian Metallothioneins Contain Copper

Table 1 summarizes the metal composition of native MTs. It shows the variable metal content of MTs and, importantly, that copper is bound in all instances where MT was not induced by zinc, albeit there remains some uncertainty as to the extent to which it is present owing to the procedures employed in isolating the protein. Remarkably, in cadmium-exposed rats, plasma MT also contains copper [56]. Moreover, the metal composition of MTs depends on age, e.g., with a linear increase of Cd^2+^ with age, and on diseases associated with zinc and copper metabolism [57,58]. The presence of Cu^+^ ions in native MTs, in particular MT3 and MT4 (Table 1), does not seem to be an artefact of the isolation of MT from tissues and suggests a role in copper metabolism. Human foetal and neonatal MTs contain significant amounts of Cu^+^ as also reported for MTs from other species [59,60]. Zinc and copper MTs are differentially distributed: CuMT can be found in lysosomes under conditions of excess copper while Zn-MT is mainly cytosolic [61]. Since the Cu–S(thiolate) bond is more thermodynamically stable as well as kinetically labile than the Zn–S(thiolate) bond, CuMT can exist under the acidic conditions in lysosomes [61]. Whether Cu^+^-only MTs co-exist with Zn^2+^-only MT is not known. In diseases associated with copper accumulation, e.g., Wilson disease, hepatic cellular carcinoma, primary biliary cirrhosis and certain animals such as the Long-Evans cinnamon rat, Bedlington terrier, and toxic milk mouse, more Cu^+^ is sequestered in MT [62]. Many tumours have a higher copper content, in particular melanoma [63].

In a murine model of Menkes disease, copper accumulates in intestinal cells and a large fraction is bound to MT. Crosses of the disease model and *Mt1/Mt2* k.o. mice revealed a high degree of copper toxicity and demonstrated a physiological function of MT in Cu^+^ sequestration [64].

## 6. Cu^+^ Affinity for Metallothionein

In the first period of 3d metals, Cu^2+^ has the strongest affinity to ligands [65]. Accordingly, its free metal ion concentration is the lowest among the divalent essential biometals. In biological systems, the situation is more complex due the fact that Cu^+^ is the preferred redox state under the reducing conditions in cells. Yet, the valence state of copper in some cellular proteins is Cu^2+^. For yeast copper superoxide dismutase, it has been estimated that the binding constant for Cu^+^, *K*_b_ about 10^20^ M^−1^_,_ is about five orders of magnitude higher than the one for Cu^2+^, *K*_b_ about 10^15^ M^−1^ [66]. Likewise, murine *S*-adenosylhomocysteine hydrolase has a *K*_b_ of 3.8 × 10^14^ M^−1^ for Cu^2+^ [67]. Thus, the cellular concentration of free Cu^2+^ is potentially higher than the one for Cu^+^ based on these equilibrium considerations but any free Cu^2+^ is expected to be reduced to Cu^+^. The high toxicity of Cu^+^ would seem to require an efficient buffer that avoids its exquisite Fenton chemistry which results in formation of reactive oxygen species. Estimates of binding constants of Cu^+^ to MT are *K*_b_ > 2 × 10^16^ M^−1^ [68]; 4.1 × 10^16^ M^−1^ [69]; and 2.1 × 10^15^ M^−1^ in case of Cu_12_MT3 [70]. This high affinity suggests that MT buffers Cu^+^ to at least femtomolar concentrations. If one considers that the volume of a typical cell is in the range of a few picoliters (10^−12^ L), such a concentration is at the single ion per cell concentrations, essentially solving the issue of how to avoid Fenton chemistry. Moreover, to avoid Fenton chemistry during transfer of copper, cellular Cu^+^ traffic is mediated by metallochaperones. In the absence of bona fide zinc metallochaperones, MT can have an active role in cellular Zn^2+^ re-distribution. Furthermore, the presence of both Zn^2+^ and Cu^+^ in MT indicates an effect of copper on zinc buffering and vice versa.

The role of human MTs in normal copper metabolism other than serving as a buffer for Cu^+^ is less clear. A rather constant Cu^2+^/Zn^2+^ ratio is maintained in serum. During inflammation and ageing, the ratio increases [71]. Extracellularly, Cu^2+^ can be scavenged and reduced by extracellular MT3 [72,73]. In MT3, bound Cu^+^ is redox-stable [74]. Different structures of MT are obtained depending on whether copper is titrated into T or into Zn_7_MT, where metal-thiolate clusters have already established a particular protein structure. The stoichiometry of seven divalent metal ions does not apply to monovalent ions. Up to 12 Cu^+^ ions can be titrated into rabbit liver Zn-MT2. They bind in one Cu_6_S_9_ and one Cu_6_S_11_ (both Cu^+^-thiolate) cluster [75]. When Cu^+^ is titrated into T, two distinct Cu_4_-thiolate clusters are formed with 12–14 cysteine residues involved in Cu^+^ ion binding [76]. The Cu_8_MT species is a stable intermediate characterized by a breakpoint in the titration following the phosphorescence of Cu^+^–S bonds, followed by the binding of an additional four Cu^+^ ions. A titration followed by ESI-MS, identified Cu_4_S_6_ and Cu_6_S_9_ clusters in the β-domain followed by formation of Cu_4_S_6_ and Cu_7_S_x_ clusters in the α-domain with clusters having specific emission and CD properties [77]. The binding of additional copper ions up to a stoichiometric ratio of 20:1 was observed. The work was performed with a human MT1A protein that contains 70 instead of the 61 amino acids of native MT1A. Clearly, these supermetallated species have no significance under normal physiological conditions as the availability of zinc and copper is controlled. Also, as discussed below, supermetallation has been observed as a result of the nature of the mass spectrometric investigation. A 3D NMR structure of the individual domains of murine MT1 demonstrates seven Cu^+^ ions bound in trigonal geometry, three in the α-domain and four in the β-domain [78]. The copper protein structure is different from that of the zinc protein. Thus, MT is a protein whose structure depends on the type of metal ion bound. A Cu_4_S_8–9_ cluster instead of the typical Zn_3_S_9_ cluster has also been observed in human MT3 [74]. There seems to be a domain preference for Cu^+^ binding. The β-domain seems to have a marginally higher affinity for Cu^+^ [77].

## 7. Zn^2+^ Affinity for Human Metallothionein

The discovery of MT and its initial metal binding characterisation soon uncovered that this small, highly cysteine-rich protein interacting with various metal ions and variable metal load in vivo is a challenging system for biochemical and biophysical investigations. One of the pioneers in the field, Bert L. Vallee, compared MT to a Sphinx, who only very slowly uncovers her deeply hidden secrets. Indeed, after 60 years of MT research and over ten thousand research articles we are just beginning to understand its biological function, which goes well beyond the originally postulated role in storage of metal ions and includes regulatory functions in zinc and copper metabolism. One of the most controversial MT parameters is its affinity for metal ions. It is critical for understanding its function. While the affinity for metal ions was investigated from the beginning, a discussion of the underlying assumptions is necessary to understand the conclusions. For many years, based on a model of cooperative metal binding it was thought that seven Zn^2+^ ions are bound in MT with identical and high affinity. Early spectroscopic studies on pH-dependent Zn^2+^ binding to MT indicated a characteristic one-step binding mode that usually supports equivalence in metal ion binding to an apo-protein [79]. Investigation of Zn^2+^ binding and characterization of the acidity of cysteines in T (average p*K*_a_^SH^ ≈ 8.9) allowed calculation of the apparent binding constant (*K*_b_) of overall identically bound Zn^2+^ ions in equine liver MT as 2 × 10^12^ M^−1^ at pH 7.0 [79]. Similarly, investigations performed in the 1980s showed that Zn_7_MT interacts with many chelators such as EDTA, NTA, H_2_KTSM or terpyridyl causing Zn^2+^ ion dissociation from the protein [80,81]. Kinetic studies of Zn^2+^ transfer to the chelators revealed two kinetic classes of Zn^2+^ ions in the protein. It has been suggested that there are seven independent metal ion sites of two kinetic types. Using these concepts, stability constants of Zn_7_MT were calculated using NTA and H_2_KTSM and competitors/chelators [81,82]. The same apparent constants (1.8 × 10^11^ M^−1^) per Zn^2+^-binding site were determined at pH 7.4, independent of whether the Zn^2+^ ions were grouped into two kinetic classes or treated as seven non-interacting sites with equal affinity for Zn^2+^. The application of differential pulse polarography (DPP) in the presence of metal chelators resulted in the determination of apparent stability constants of 1.3 × 10^12^ and 7.9 × 10^11^ M^−1^ in the case of rabbit MT2 and MT1, respectively [83]. Equilibration between MT and the calcium chelator 5F-BAPTA, which also binds Zn^2+^ efficiently, has been used in ^19^F-NMR spectroscopy to determine apparent Zn^2+^ binding constants of rabbit MT2 and human MT3 as 3.1 × 10^11^ and 6.2 × 10^10^ M^−1^, respectively [84]. In all these physicochemical approaches, one critical assumption requires scrutiny in order to understand the limitation of the conclusions and what is needed to obtain an accurate description of the system: Seven Zn^2+^ ions always were considered to be thermodynamically equivalent and any possible difference in the stability of the seven Zn^2+^ ions was not resolved. The pH-dependent Zn^2+^ binding and competition under this assumption are given in Equations (1) and (2).

apo-MT + 7Zn^2+^ ⇆ Zn_7_MT (1)

Zn_7_MT + chelator ⇆ apo-MT + 7Zn-chelator (2)

Noteworthy, the existence of two species of the same protein, T and MT, was explained early on by a high positive cooperativity of Zn^2+^ binding to the cysteine donors [68]. Cooperativity was also inferred from spectroscopic observations [79]. ^113^Cd-NMR spectra were interpreted in terms of positive cooperativity when the first couple of Cd^2+^ ions bind to the protein and NMR signals appear simultaneously [32,79]. In case of Zn^2+^ ions, no method existed to examine cooperativity in binding. Positive cooperativity implies that the binding of the first metal ion (first event) promotes the binding of the remaining metal ions that would bind with higher affinity. If binding were to occur with high positive cooperativity as proposed originally for ZnMT, the protein would buffer Zn^2+^ only in an extremely narrow range. The current knowledge about binding constants seems to rule out such positive cooperativity. Four Zn^2+^ ions seem to bind with similar, high affinity and what may appear as cooperativity is a consequence of the metal determining protein structure for binding the additional metal ions in a clustered arrangement [30]. The association of the remaining three Zn^2+^ ions occurs entirely in a sequential mode [30]. Such characteristics make MT a very good zinc buffer with a wider range of pZn buffering as indeed necessary to cover the range of changes of cellular free Zn^2+^ ions.

The original assumption of all seven metal ions being thermodynamically equivalent in binding introduces a major error by neglecting the possibility of more weakly bound metal ions. To demonstrate how this bias occurred, one may consider two simple examples. In the first one, the affinities of all seven metal ions are equal (log*K*_b_ = 12.0) and the average binding constant of 12.0 applies to each of them. In the second example, six metal ions are bound with the same high affinity of 12.0 but one ion is bound with significantly lower affinity with a log*K*_b_ value of 9.0. In this case, the assumption of equivalence of all seven metal ions results in an average log*K*_b_ value of 11.6, which is very similar to the value of 12 in the first example, and not readily resolved by the methods employed in the past. The average value does not reflect the more complex character of the protein in terms of metal binding and does not indicate the presence of one metal ion bound with nanomolar affinity only.

Furthermore, in spectroscopic pH-titrations, it was assumed that all cysteine thiol residues have the same p*K*_a_ values, a scenario that is unlikely due to the differences in chemical environments of the cysteines in the protein. The exact difference in acidity of the sulphur binding ligands cannot be observed in UV-titrations due to the presence of 20 cysteines and overlapping dissociation events (macroscopic vs microscopic p*K*_a_) [85,86].

Recent studies suggest that T, in particular the form one obtains when MT is demetallated, is not in a random coil conformation and therefore there is the possibility that local structure influences the acidity of particular cysteine residues [87,88]. We showed that cysteines of certain CXXC motifs in zinc binding sites possess significantly different acid-base properties due to the presence of S⋯H–N hydrogen bonds [36,89]. Such hydrogen bonds exist in MTs [9,90,91] and could be responsible for the observed structure of T despite the apparent lack of a defined overall structure of the protein. The differences in acidity of cysteine thiols result in various affinities of the particular Zn^2+^ ions at neutral pH. Therefore, assuming that the incremental increase of absorbance at ~220 nm due to S^−^ → Zn^2+^ charge transfer is linear and identical for each Zn–S bond in pH-dependent titrations is not appropriate. In addition, the exact Zn^2+^ association and dissociation pathways in MT are unknown and variable fractions of terminal and bridging sulphur donors per Zn^2+^ ion during the binding processes make the direct spectroscopic titration method inconclusive and prone to error when attempting to determine the affinities of seven chemically different Zn^2+^ ions. The application of competing chelating reagents to determine the fraction of Zn^2+^ transferred during equilibration, such as in the case of 5F-BAPTA and MT, is currently a standard procedure in metal affinity determination of metalloproteins if the stability constant of the competing reagent is known under the condition of the experiment [92,93]. However, different constants are obtained when various binding models are considered. Assuming thermodynamic equivalence of seven Zn^2+^ ions results in an apparent Zn^2+^ binding constant of ~10^11^ M^−1^ [81]. Remarkably, the use of NTA for the competition with Zn_7_MT2 at various NTA/MT ratios showed high Zn^2+^-to protein affinity and equivalence of Zn^2+^ ions when NTA was used at high reagent/protein molar ratios [30]. Decreasing the NTA concentration resulted in higher Zn^2+^ transfer from MT than what is expected if all seven Zn^2+^ ions were bound with the same affinity. This observation indicates differences in the affinity of the Zn^2+^ ions with an additional indication that at least one Zn^2+^ ion binds with lower affinity to MT. The existence of more weakly bound Zn^2+^ ions in MT has been shown in several publications using enzymatic assays. The incubation of Zn_7_MT2 with the apo-forms of sorbitol dehydrogenase (SDH) and carboxypeptidase A caused rapid recovery of enzymatic activity due to fast transfer of ~1 Zn^2+^ ion from MT [51,94,95]. Longer incubation of MT2 with apo-SDH at various ratios demonstrated that more than one Zn^2+^ ion can be transferred from MT [51], consistent with transfer of the more tightly bound Zn^2+^ ions. However, SDH reconstitution with Zn^2+^ is significantly more efficient than expected based on a Zn^2+^ affinity of *K*_b_^SDH^ = 1.6 × 10^11^ M^−1^ and the assumed presence of only tightly bound Zn^2+^ ions in MT with an overall apparent binding constant of ~10^12^ M^−1^ [51]. All the above mentioned methods and enzymatic assays demonstrated that three classes of Zn^2+^ ions are present in MT: strongly bound Zn^2+^ with picomolar affinity, a weakly bound Zn^2+^ ion that exchanges fast, and some Zn^2+^ ions with high picomolar affinity and transferred significantly slower to Zn^2+^ acceptors. The exact affinity values of the weakly, i.e., with moderate affinity, bound Zn^2+^ ions were not determined by enzymatic assays with recipient proteins. However, recovery of activity of the apo-form of alkaline phosphatase [95], which binds two Zn^2+^ ions with binding constants of ~10^8^ M^−1^ [96], and Zn^2+^ inhibition of protein tyrosine phosphatase 1B with an apparent binding constant of 6.3 × 10^7^ M^−1^ [51] clearly demonstrate the presence of a weakly bound Zn^2+^ ion with nanomolar affinity in MT.

Besides spectroscopic and enzymatic methods and assays, electrospray mass spectrometry provided new opportunities for determining metal ion affinity for MT. In the early literature on the subject, it was reported that the relative abundances of various complexes in the samples can be estimated from abundances of respective ions in the ESI-MS spectrum [97,98]. The application of this method for Zn^2+^ metallation in several MT isoforms (MT1–3) shows that metal ion binding to the protein is sequential rather than cooperative and several Zn_1–7_MT species are observed in mass spectra at various Zn^2+^ to T ratios [99,100,101,102]. This observation is in contrast to the assumed equivalent affinity of all seven Zn^2+^ ions as well as positive cooperativity in binding and supports the above conclusion about the various affinities of zinc sites in MT. Zn^2+^ binding to human apo-MT1A (product of the expression was a 72 amino acid protein instead of 61) was studied by ESI-MS in the presence of apo-CA (carbonic anhydrase) as a Zn^2+^ competitor with a known high affinity for Zn^2+^ [102]. Using the intensity of particular Zn_1–7_MT1A, apo-CA, and Zn-CA species from mass spectra and the known Zn-CA stability (*K*_b_^CA^ = 2.5 × 10^11^ M^−1^) [103], it was concluded that Zn^2+^ transfer to CA is sequential and that the apparent binding constants of the seven Zn^2+^ ions (*K*_b1_–*K*_b7_) vary from 3.2 × 10^12^ to 6.3 × 10^11^ M^−1^. Although the method seems elegant, it is only qualitative. Our results of Zn^2+^ binding to T performed in the presence of Zn^2+^ donors with various affinities (unpublished data) demonstrate that metal binding analysis performed by ESI-MS does not assess completely the equilibria present in solutions. Two recent papers published in mass spectrometry journals prove that investigations of Zn^2+^-equilibria by electrospray ionization in the gas phase are not quantitative due to zinc deposition or protein supermetallation during ESI-MS analysis [104,105].

Importantly, complicated systems with several metal ions bound to the protein should be characterized using different methods in order to overcome the limitations of a single method. The methods need to have the power to resolve small differences. When competitors are used to determine Zn^2+^ affinity, their dynamic range for detecting changes should be taken into account. In typical competition experiments, Zn^2+^ transfer is measured when the competitor is saturated—ideally in the range of 20–80% or at least 10–90%. Depending on the (spectroscopic) detection mode and the sensitivity one cannot determine quantitatively saturation changes below 10% due to low signal to noise ratios. This automatically limits the range of affinities that can be determined with a particular competitor to a binding constant of only one order or magnitude higher than the Zn^2+^ binding constant of the competitor. If the protein binds more than one Zn^2+^ ion with potentially different affinities, competitors with a larger dynamic range should be employed. Alternatively, a series of competitors with various affinities toward Zn^2+^ can be employed.

We employed the extremely sensitive zinc fluorescent probe FluoZin-3 for the detection of Zn^2+^ transfer from human MT2 [30]. The linear dynamic range of FluoZin-3 is very large and the high sensitivity of fluorimeters allows the detection of picomolar concentrations of Zn^2+^. The metal-free probe is almost non-fluorescent and its saturation by Zn^2+^ increases fluorescence by about 25,000 times when fully saturated [106]. Although FluoZin-3 binds Zn^2+^ with an apparent binding constant of 1.1 × 10^8^ M^−1^ [23,107] its application allows measuring Zn^2+^ below 0.01% of Zn^2+^ saturation due to its optical properties and the sensitivity of the fluorescence measurements. Thus, one can measure low picomolar affinities (~10^12^ M^−1^) when using micromolar concentrations of the probe. The system is fully reversible. Zn^2+^ binding to T in the presence of the probe and Zn^2+^ dissociation from Zn_7_MT2 and transfer to FluoZin-3 results in the same coordination mode and metal ion affinity. The most significant outcome of the investigation was the quantitative determination of three classes of Zn^2+^ affinities in MT. Four Zn^2+^ ions bind with high affinity of *K*_b1–4_ ≈ 10^12^ M^−1^ with apparent high cooperativity of Zn^2+^ binding in forming the Zn_4_MT species [30]. Another two Zn^2+^ ions bind to the protein with lower 10^10^–10^10.5^ M^−1^ (*K*_b5_, *K*_b6_) affinity. And the seventh Zn^2+^ ion, regardless of whether one measures association or dissociation, binds with nanomolar affinity (*K*_b7_ ≈ 10^8^ M^−1^). The same values were determined when the slightly less sensitive fluorescent chelating agent RhodZin-3 was used. The species Zn_6_MT could be involved in buffering zinc under conditions of zinc signalling where low nanomolar concentrations of zinc are reached transiently. Under these conditions Zn_7_MT could be formed.

**Figure 2:**
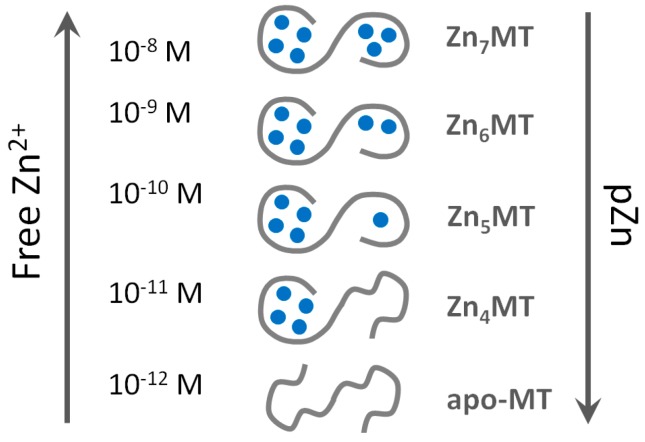
Structural polymorphism of human zinc metallothionein as a function of cellular free Zn^2+^ (pZn) concentrations.

This finding changed completely the understanding of how MT functions. Instead of being a thermodynamic sink for Zn^2+^ as postulated previously (based on a high cooperativity model), it allows MT to participate actively as a donor and acceptor of Zn^2+^ in the cell (Figure 2). The presence of a more weakly bound Zn^2+^ ion in MT became the basis of some controversy but we believe our methodology is sound and not fraud with the limitations of other methods used for the study of Zn^2+^-metallothionein interactions [30]. A recent article on the thermodynamics of Zn^2+^ and Pb^2+^ interacting with MT3 is remarkable in this regard [108]. Using isothermal titration calorimetry (ITC) and competition of EDTA with Zn_7_MT3, exactly the same three classes of Zn^2+^ affinities were found. Although the study was performed at pH 6.8 due to Pb^2+^ precipitating at higher pH, it was found that four Zn^2+^ ions are bound with an overall apparent binding constant of 7 × 10^10^ M^−1^ and another two with 2 × 10^9^ M^−1^. The seventh Zn^2+^ ion was found to bind with a constant of 7 × 10^7^ M^−1^. Adjusting those constants to the pH of 7.4 used in our studies with FluoZin-3, not only the same stoichiometry but also almost identical affinities of Zn^2+^ in MT3 compared to MT2 pertain. Moreover, Pb^2+^ ions bind to MT3 with the same stoichiometry as Zn^2+^ with the classes for moderate and more weakly bound ions demonstrating higher affinity relative to Zn^2+^.

## 8. Metallothionein: A Metamorphic Protein with a Structure Depending on Metal Load and Redox State

Regardless of the method used to investigate affinities of MT for Zn^2+^, the most important conclusion for the function of the protein is that seven Zn^2+^ ions in fully saturated MT are in tetrathiolate coordination environments but not bound with equal affinities. Particular Zn^2+^ ions differ by several orders of magnitude in their affinity towards MT. The consequence of such a behaviour of the protein is its inherent heterogeneity in vivo in terms of metal loading and the existence of the protein in several forms: partially metallated states when there is not enough Zn^2+^ to saturate the protein (less than seven metal ions). The redox biology of the protein increases further the number of such states. These findings provide an entirely different description of the protein structure from the one considered dogmatic for so many years and based on identical affinities of all Zn^2+^ ions, namely that the protein exists only as either T or fully saturated Zn_7_MT. A wrong model was accepted for a long time in the absence of additional information regarding metallothionein’s function in metal and redox metabolism, the signalling roles of Zn^2+^ ions, and the control of cellular zinc and copper. The application of ultrasensitive fluorescent probes and sensors for the measurement of cellular free Zn^2+^ concentration at the ~10^−10^ M level has changed the field as it established regulatory functions of Zn^2+^ [37]. MT with seven Zn^2+^ ions bound with ~10^12^ M^−1^ affinity is incompatible with handling free Zn^2+^ ions at concentrations varying from 10^−8^ to 10^−11^ M. Our investigations with the human HT-29 colon cancer cell line demonstrated that cells under various physiological states have a surplus of tight-binding zinc ligands of about 10% over total cellular Zn^2+^ [23]. We also showed that MT is the most important component of the surplus of ligands for metals, indicating a crucial role in cellular zinc buffering [23,109]. MT can buffer Zn^2+^ due to its varying affinities for Zn^2+^ (from ~10^8^ to 10^12^ M^−1^), which remarkably overlap with cellular transients in Zn^2+^ concentration. The simple presentation in Figure 2 based on known affinities of human MT2 for Zn^2+^ demonstrates that MT does not exist as the Zn_7_MT form under normal physiological condition but that Zn_6_MT and even Zn_5_MT species are the most representative structures of cellular MT.

The existence of MT in not fully metal-loaded forms explains its buffering properties and its functions as both a Zn^2+^ donor and a Zn^2+^ acceptor. Several investigations on Zn^2+^ transfer showed that a mixture of Zn_7_MT and T, which in fact is the mixture of partially saturated species (Zn_4–6_MT) serves as a donor for Zn^2+^ activating for example apo-metalloenzymes or inhibiting non-metalloenzymes such as protein tyrosine phosphatase 1B, depending on prevailing Zn_7_MT to T ratios [51]. Clearly, this discussion focused on mammalian MTs and human MTs in particular. The metal binding characteristics and redox properties of MTs in non-mammalian species need to be determined and related to the specific features of zinc and copper metabolism in these species. Such investigations are expected to lead to a genuine understanding of the varying structures of the fascinating metal cluster in the different MT families in relation to biological function.

Our investigations show that besides redox activity [20], the most important property for zinc and copper metabolism is a function of MT as metal buffer under physiological conditions. MT in its fully metal-loaded state in the 3D structures established by NMR spectroscopy and X-ray diffraction cannot serve regulatory and signalling function of Zn^2+^ ions. The available 3D structures of MT represent only a partial picture of the multiple MT structures present in the cellular environment. The other partially saturated forms, for which structures with zinc are not known, constitute the complementary forms required for satisfying the buffering properties of MTs. Several reports showed already that the protein is present in tissues as a species that is not saturated with zinc and that the level of saturation differs and depends on tissue localization and both physiological and pathological states. One needs to recall that analytical methods for detection and quantitative analysis of partially metal-saturated structures of individual MTs in vivo are not available. Thionein per se cannot be present in any tissue when Zn^2+^ is present. Freely available zinc binding sites in Zn_4–6_MT species are responsible for the protein frequently referred to as T.

One challenging issue for the future is the analysis of MT species in cells and tissues. One possibility is to use antibodies towards particular species. However, the metamorphic nature of the protein with structures depending on metal load and oxidation state and different structures of Zn_4–6_MT and high coordination dynamics of Zn^2+^ ions in the protein may prevent the development of such antibodies [110,111]. Another possibility are mass spectrometry techniques with combined elemental and molecular resolution [26,112,113].

## Figures and Tables

**Table 1:** Metal composition of native mammalian metallothioneins (MTs).

Formula	Source	Reference
Zn, Cd, Cu	horse kidney	[1]
Cd_0.2_Cu_0.1_Zn_6.7_MT	human liver	[25]
CuZn_6_MT	human corneal epithelial cells	[26]
Zn_7_MT	same cells, induction with Zn^2+^	[26]
Cu_4_Zn_3_MT3	human brain	[5]
Cu_2_Zn_5_MT4	mouse tongue *	[6]
Cd_2_Zn_5_MT2	rat liver, induction with Cd^2+^	[9]
Cd_3_Cu_3_ZnMT2	mouse liver, induction with Cd^2+^	[27]
Cd_4_CuZn_2_MT1	mouse liver, induction with Cd^2+^	[27]
Cd_6_CuMT1/MT2	mouse liver, induction with Cd^2+^	[27]

* Based on a measured metal ratio of 2.6 and an assumed stoichiometry of seven bound divalent metal ions.

**Table 2:** Human metallothionien isoforms UniProtKB/Swiss-Prot, annotation score (from 0 to 5), information on protein or transcript level, and natural protein variants. Entry number of MT4 is provided from the NCBI data base due to the wrong protein sequence deposited in UniProt (MT4_HUMAN, P47944), which refers to an MT4 variant (30C → Y, R31 → W).

MT Isoform	Number of Amino Acids	UniProt Name	Entry Number	Annotation Score	Protein Existence *	Variants
MT1A	61	MT1A_HUMAN	P04731	5	PL	T27 → N, K51 → R
MT1B	61	MT1B_HUMAN	P07438	4	PIH	-
MT1E	61	MT1E_HUMAN	P04732	5	PL	-
MT1F	61	MT1F_HUMAN	P04733	5	PL	-
MT1G	62	MT1G_HUMAN	P13640	5	PL	A10 deletion (isoform 2)
MT1H	61	MT1H_HUMAN	P80294	5	PL	-
MT1L (MT1R)	61	MT1L_HUMAN	Q93083	3	TL	-
MT1K (MT1M)	61	MT1M_HUMAN	Q8N339	4	PIH	T20 → K
MT1X	61	MT1X_HUMAN	P80297	5	PL	-
MT2	61	MT2_HUMAN	P02795	5	PL	A42 → V
MT3	68	MT3_HUMAN	P25713	5	PL	-
MT4	62	Metallothionein 4	AAI13443.1	3	PIH	30C → Y, R31 → W, G48 → D

* PL: experimental evidence at protein level; PIH: protein inferred from homology; TL: experimental evidence at transcript level.

**Table 3:** Zinc-dependent transcription factors for human *MT1* genes. The numbers designate *cis* acting elements in the promoters of the different *MT1* genes [40].

Transcription Factor *	Sp1	MTF1	EGR1	GR	RAR	Ikaros	Churchill
**Zinc Motif**	**ZnFinger**	**6ZnFingers**	**ZnFinger**	**ZnTwist**	**ZnTwist**	**ZnFinger**	**ZnFinger/ZnCluster**
*MT1*							
A	5	2	4	1	2	2	1
E	1	1	3	6	1	2	6
J (pseudogene)	2	2	3	1	3	4	8
B	1	4	-	1	5	8	3
K/M	4	2	8	2	2	2	9
G	5	2	-	-	3	6	6
F	8	5	5	-	5	6	8
H	2	5	-	3	3	7	5
X	3	3	4	2	3	9	5

* Sp1: specificity protein 1; MTF1: metal-response element (MRE) binding transcription factor 1; EGR1: early growth response protein 1 (also known as Zif268 (zinc finger protein 225) or NGFI-A); GR: glucocorticoid receptor; RAR: retinoic acid receptor; Ikaros: IKZF1; Churchill: chch.

## Abbreviations

NTA	Nitrilotriacetic acid
BAPTA	1,2-Bis(2-aminophenoxy)ethane-*N*,*N*,*N*′,*N*′-tetraacetic acid
H2KTSM	3-Ethoxy-2-oxobutyraldehyde-bis(*N*^4^-methylthiosemicarbazone)
